# Unsymmetrical cyanine dye via in vivo hitchhiking endogenous albumin affords high-performance NIR-II/photoacoustic imaging and photothermal therapy

**DOI:** 10.1186/s12951-021-01075-0

**Published:** 2021-10-24

**Authors:** Pengfei Xu, Linan Hu, Cheng Yu, Weidong Yang, Fei Kang, Mingru Zhang, Pei Jiang, Jing Wang

**Affiliations:** 1 Department of Nuclear Medicine, Xijing Hospital, Fourth Military Medical University, #127 West Changle Road, Shanxi 710032 Xi’an, People’s Republic of China; 2 Institute of Clinical Pharmacy and Pharmacology, Jining First People’s Hospital, Jining Medical University, Jining, 272000 People’s Republic of China; 3 Departments of Radiology, The Second Xiangya Hospital, Central South University, Changsha, Hunan 410011 People’s Republic of China

## Abstract

Herein, an unprecedented synergistic strategy for the development of high-performance NIR-II fluorophore is proposed and validated. Based on an unsymmetrical cyanine dye design strategy, the NIR-II emissive dye NIC was successfully developed by replacing only one of the indoline donors of symmetrical cyanine dye ICG with a fully conjugated benz[c,d]indole donor. This minor structural change maximally maintains the high extinction coefficient advantage of cyanine dyes. NIC-ER with endogenous albumin-hitchhiking capability was constructed to further enhance its in vivo fluorescence brightness. In the presence of HSA (Human serum albumin), NIC-ER spontaneously resides in the albumin pocket, and a brilliant ~89-fold increase in fluorescence was observed. Due to its high molar absorptivity and moderate quantum yield, NIC-ER in HSA exhibits bright NIR-II emission with high photostability and significant Stokes shift (>110 nm). Moreover, NIC-ER was successfully employed for tumor-targeted NIR-II/PA imaging and efficient photothermal tumor elimination. Overall, our strategy may open up a new avenue for designing and constructing high-performance NIR-II fluorophores.

**Supplementary Information:**

The online version contains supplementary material available at 10.1186/s12951-021-01075-0.

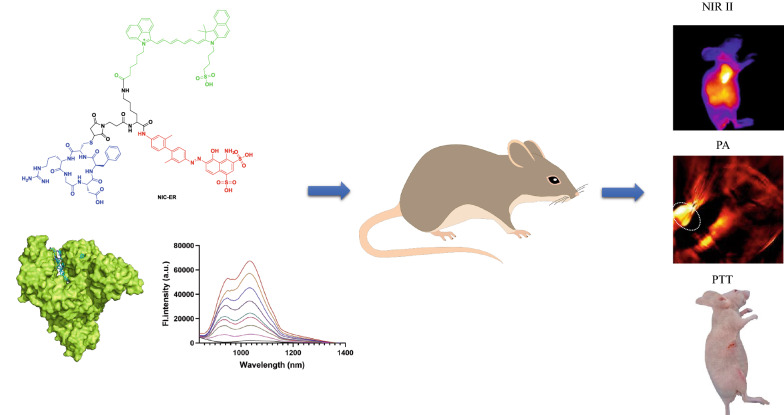

## Introduction

Recently, optical imaging in the “second near-infrared (NIR-II) window” (1000-1700 nm) has been considered as an ideal imaging modality than traditional NIR-I (650-900 nm) imaging [1–3]. So far, the most active materials used for NIR-II imaging and therapy are mainly nanoparticles (single-walled carbon nanotubes and lanthanide nanoparticles) and quantum dots [4–7]. In terms of clinical translation ability, small organic fluorophores offer a better alternative due to their compact molecular structure, minimal toxicity, and facile derivatization [8–16]. Interestingly, typical NIR I fluorophore ICG, one of the most famous cyanine dyes, can be used for NIR-II imaging by exploiting the off-peak emission beyond 1000 nm [8, 17–20]. However, the off-peak emission of ICG maybe not be bright enough for deep tissue NIR-II imaging. To achieve longer emission, conjugated donor groups (benz[c,d]indole and indolizine) have been introduced into the cyanine bridge to delocalize the frontier molecular orbitals further. For example, replacing the indoline donor of typical indoline cyanine dyes with a fully conjugated benz[c,d]indole donor results in a red-shift emission of above 200 nm [21]. In another case, heptamethine cyanine dye FD-1080 uses two benz[c,d]indoles as terminal heterocycles affording maximal emission wavelengths over 1000 nm, although this come at the expense of decreased molar extinction coefficient (2.9 × 10^4^ M^−1^ cm^−1^ vs. 2.3 × 10^5^ M^−1^ cm^−1^ of ICG) [22]. Although NIR-II fluorescence imaging has achieved encouraging results in tumor imaging and angiography, most of the reported organic NIR-II fluorophores suffer from low molar absorptivity, complicated synthesis steps, low aqueous solubility, or poor quantum yields [23–25]. There is still long way to go before NIR-II dyes are ready for clinical applications.

In the present work, we apply a novel design strategy to develop a nonsymmetrical NIR-II emitting cyanine dye, where only one of the indoline donors of the typical symmetrical cyanine dye ICG was replaced by a fully conjugated benz[c,d]indole donor. This minor structure change extending the π-conjugation of the dye allows its emission red-shift into the NIR-II region while maximally maintaining the advantage of the high extinction coefficient of the parent dye (ICG). According to energy-gap law, NIR-II emissive dyes are inherently low fluorescence quantum because the generation of NIR-II emission photons requires low energy bandgap; thus, inducing non-radiative decay processes tend to dominate over radiative photon emission [26]. One method of reducing non-radiative deactivation is intercalation of organic dyes comprising large, hydrophobic π-systems into plasma proteins to restrict their conformational freedom [27, 28]. As stated in previous report, CH-4T exhibits a 110-fold fluorescence increase after forming dye-protein molecular complexes by incubation with FBS at 70 °C for 10 min [29]. Another promising method is to link NIR-II dyes with an albumin binder so that the conjugate can bind to circulating endogenous albumin in situ after intravenous injection. Evans blue (EB) is a non-toxic azo dye with high serum albumin binding affinity and excellent hydrophilicity [30, 31]. More recently, a maleimide-modified truncated Evans Blue (tEB) was developed as an “add-on” molecule for a variety of bioconjugation applications in diseases imaging and treatment [32, 33]. We hypothesized that the conjugation of NIR-II dye with tEB could afford the conjugate with endogenous albumin hitchhiking capability, which would increase its fluorescence brightness and extend its half-life in the blood [34–36]. Besides, slow release of the NIR-II emissive conjugate over time would allow continual uptake at the biological target [37, 38]. To the best of our knowledge, this designing strategy for high-performance NIR-II fluorophores has never been reported [39, 40].

Based on the above molecular design principle, we develop a novel unsymmetrical cyanine dye NIC. To impart NIC with albumin-binding and active-targeting abilities, an albumin-binding motif tEB (truncate Evans blue) and an active targeting ligand c(RGDfc) were introduced into the molecular structure of NIC (termed as NIC-ER). Compared to benchmark ICG, NIC exhibits red-shifted absorption and emission with dichromic properties. NIC-ER was easily loaded into the pocket of albumin in the presence of albumin, which induces a dominant emission at 1030 nm and a brilliant ~89-fold increase in fluorescence brightness. The absolute quantum yield of NIC-ER in HSA (Human serum albumin, 25 mg/mL) was measured to be 3.56 %, which is higher than most reported organic NIR-II dyes [45–47]. The NIC-ER in HSA exhibits bright NIR-II emission with high photostability and significant Stokes shift (>110 nm) due to the high molar absorptivity and moderate quantum yield. Then, noninvasive imaging of small blood vessels at the limb, cerebral, and abdomen region is achieved with a high signal-to-background ratio and deep penetration. Furthermore, NIC-ER has also been successfully applied for targeted tumor imaging (NIR-II fluorescence/PA synergistic imaging) and efficient tumor elimination (photothermal therapy). In summary, this study offers a superior NIR-II cyanine dye with satisfying brightness, large Stokes-shift, and high photostability and opens up a new avenue for designing and constructing high-performance NIR-II fluorophores.

## Results and discussion

As shown in Additional file 1: Scheme S1, NIC and NIC-ER were easily synthesized. The synthesis began with the heterocyclic salts and *N*-[5-(phenylamino)-2,4-pentadienylidene] aniline monohydrochloride. This reaction proceeded well in 58 % yield affording NIC. NIC-MLEB was prepared through the direct conjugation of the carboxylic acid group of NIC with the compound MLEB. Then, the free thiol group of c(RGDfc) was covalently attached to the maleimide motif of NIC-MLEB to produce the final product NIC-ER.

**Fig. 1:**
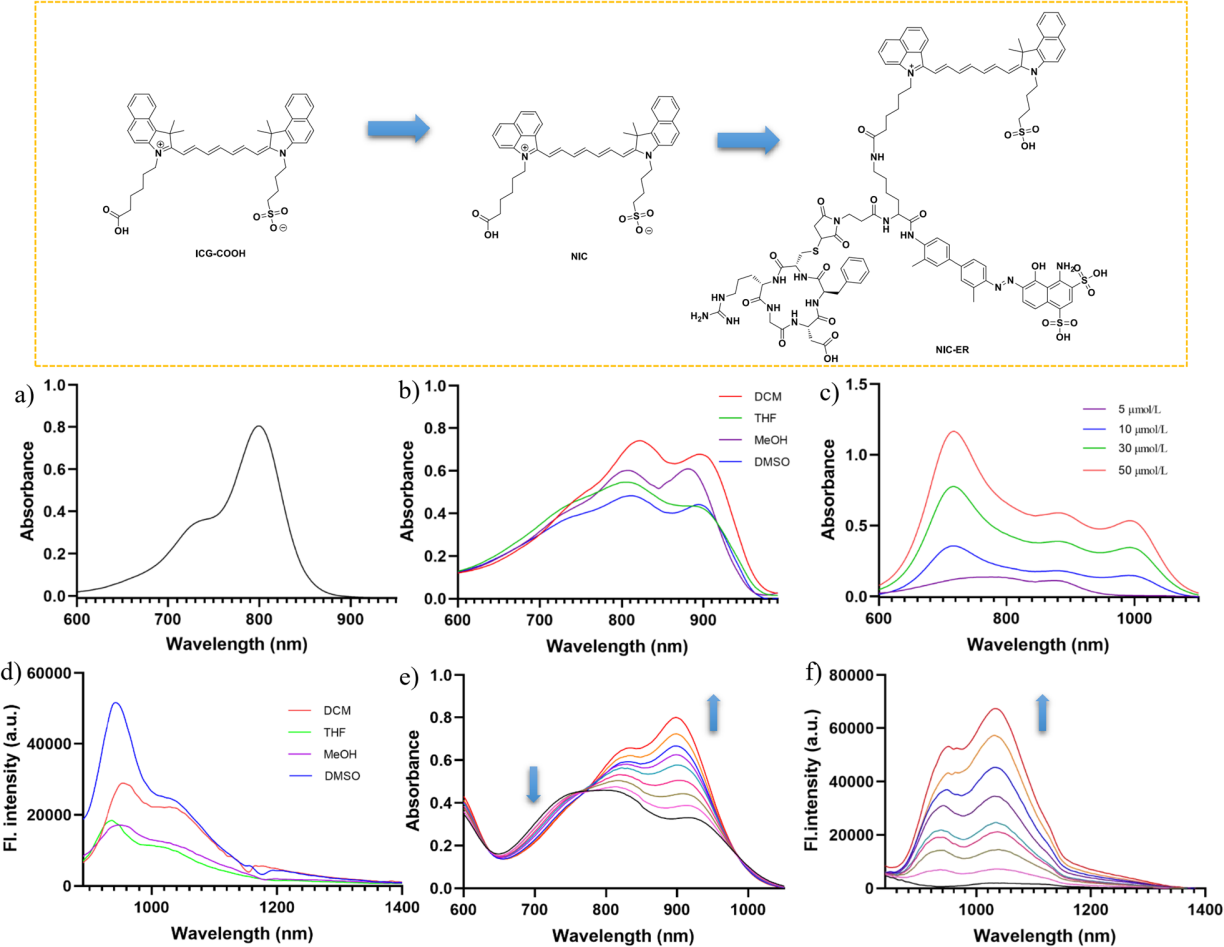
Chemical structures of the molecular fluorophores (ICG-COOH, NIC and NIC-ER) and their optical properties. **a** Absorbance spectrum of ICG-COOH in DMSO. **b** Absorbance spectra of NIC in different solvents. **c** Absorbance spectra of NIC in PBS with different concentrations. **d** Emission spectra of NIC in different solvents. **e** Absorbance spectra of NIC-ER in PBS added with different concentrations of HSA from 0 mg/mL to 25 mg/mL. **f** Emission spectra of NIC-ER in PBS added with different concentrations of HSA from 0 mg/mL to 25 mg/mL

With NIC in hand, its spectroscopic properties were first studied in various solvents. Typical to cyanine dyes, NIC exhibits a maximum absorption peak with a blue-shifted shoulder, even though the shoulder has a relatively enhanced contribution in the overall absorption spectrum. Figure 1b shows that the solvents had a significant effect on the absorption curve shape of NIC. DMSO shows the closest ratio of high-energy to lower-energy features nearing equal heights. High-energy absorption is the dominant feature in THF and DCM, while low-energy absorption is dominant in MeOH. The molar absorptivities of NIC differ from 1.3 × 10^5^ to 2.0 × 10^5^ M^−1^ cm^−1^ depending on the choice of the solvent. Due to planar π-electron conjugation systems, typical cyanine dyes tend to self-aggregate in aqueous buffer solutions, forming two different structures (J-aggregations and H-aggregations) depending on the angle of slippage (α) [41]. This phenomenon was also observed in this study. When NIC was directly added to PBS (high concentrations, ≥ 5 × 10^−6^ M), H-aggregates with an absorption band blue-shifted to 700 nm and J-aggregates with absorption band red-shifted to 1000 nm were observed simultaneously (Fig. 1c).

**Fig. 2:**
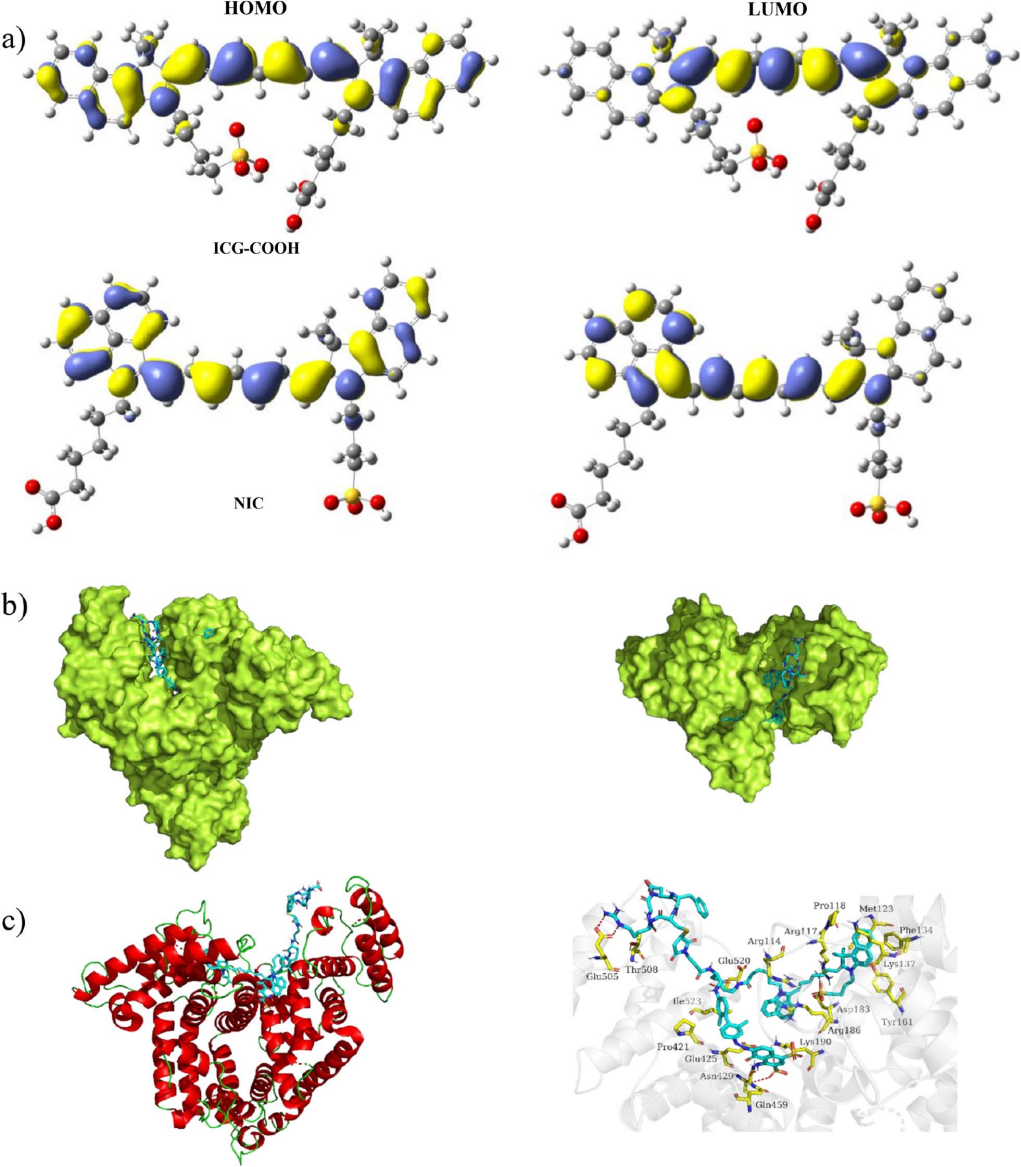
**a** HOMO and LUMO wave functions in the geometrically optimized structures of ICG-COOH and NIC. **b**–**c** The possible interaction modeling between NIC-ER and albumin. **b** Docking modeling for the NIC-ER @albumin complex. **c** Details of binding residues and active pocket between NIC-ER and albumin

Compared to the reference ICG-COOH with an absorption maximum at 800 nm, there is approximately a 100 nm red-shift, which can be tentatively ascribed to the further conjugated electron-deficient system in the benz[c,d]indole heterocycle. Density functional theory calculation was conducted (Fig. 2a, b) to examine the geometric and electronic properties of NIC and ICG-COOH. For ICG-COOH, the HOMO is uniformly distributed between the indoline nucleus ring and the polymethine chain systems, while the LUMO is mostly on the polymethine chain. The HOMO and LUMO are more extended to the conjugated benz[c,d]indole heterocycle in NIC, which would maximize the π-orbital overlap and oscillator strength. Furthermore, this decreases the HOMO-LUMO gap, which is consistent with the experimentally observed bathochromic shift in absorption.

The emission curve shapes of NIC variation with solvents are shown in Fig. 1d. All test solvents show an emission peak at 950 nm and a shoulder at 1030 nm, and the ratio of the two emissions varied with solvents. DMSO represents the highest ratio of high-energy to low-energy peak at about 2, while the remaining solvents all exhibit a ratio of 1.4 or less. No obvious emission peak was observed upon dissolution of NIC in PBS, which could be plausibly attributed to the strong excitonic interaction in the aggregates. In contrast to the high aggregation propensity of NIC, NIC-ER exhibited a dramatically decreased degree of aggregation at the same concentration in PBS. After mixing free NIC-ER dye with HSA, the truncate Evans blue motif exerts its albumin-binding effect, and this binding model induces a drastic change of absorption curve shape. As the level of HSA content increased, the intensity of the absorption peak and shoulder both increased, but with different enhancement levels. Figure 1e shows that the absorption at 900 nm possesses more enhancement relative to shorter wavelength absorption (800 nm), and the 910/800 nm absorption ratio changed from 0.8 (PBS) to 1.3 (25 mg/mL HSA). Furthermore, a similar enhancement trend was also observed in the emission data. Figure 1f shows that after mixing free NIC-ER dye with HSA, both the smaller emission peak at 950 nm and higher emission peak at 1030 nm enhanced, and a drastic ~89-fold increase in fluorescence was observed. With the addition of HSA, a longer emission peak at 1030 nm gradually increased the contribution to the overall fluorescence spectra. In the solution containing 25 mg/mL HSA, the 1030/950nm emission ratio increased to 1.25.

Even though it is well known that intercalation of dyes into the HSA pocket can lead to emission enhancement due to two events: disruption of aggregation and constrained geometry, which reduces non-radiative decay [33, 42]. However, this cannot explain why the emission peaks of NIC-ER (950 nm and 1030 nm) have different enhancement levels in the presence of HSA. From the resonance perspective, unsymmetrical cyanine dyes are characterized by two nonidentical extreme resonance configurations with different energy, which may be responsible for dichromic absorption and emission [43]. Unlike the symmetrical ICG in which the positive charge is delocalized in the entire fluorophore, the structure of NIC raises a possibility that the positive charge could be preferentially localized on one of the heterocycle N atoms in the ground state. Undoubtedly, the more stable configuration is the one in which the nitrogen of the more basic nucleus is quaternary [44]. Thus, the relative contribution of these two resonance forms determines the ratio of the two peaks in dichromic absorption in dichromic emission. We performed docking modeling to investigate the positions and interaction between NIC-ER and albumin (Fig. 2c, d). The binding site of NIC-ER was identified as the center cleft of albumin, which is consistent with a previous study that the cleft on albumin is the preferred binding site of truncate Evans blue [38]. In the binding pocket, the positively charged benz[c,d]indole heterocycle could further be stabilized by the nearby amino acids through electronic coupling and inductive effects, inducing the dominant absorption and emission at a longer wavelength range.

Besides, the absolute quantum yield of NIC-ER in HSA (25 mg/mL) was measured to be 3.56 %, which is higher than most reported organic NIR-II dyes [45–47]. Importantly, NIC-ER has satisfied molecular brightness with great potential for efficient bioimaging. Photostability and biocompatibility of NIR-II dyes are also crucial for their biomedical applications. The cellular toxicity of NIC-ER was evaluated using a CCK-8 assay (Additional file 1: Figure S1). The cellular viability of healthy human cervical cancer cells (Hela) shows a survival rate of above 90 % at the concentration of 0–50 µmol/L, suggesting the low toxicity of NIC-ER. In vivo toxicity of NIC-ER was performed after intravenous injection at 5 mg per kg mouse (10 times than the imaging doses). No significant changes were found in main organs (heart, liver, spleen, lung, and kidney) after 7 days post-injection (Additional file 1: Figures S7), suggesting low in vivo toxicity of NIC-ER. Additional file 1: Figure S2 shows that NIC-ER in HSA exhibits better photostability than ICG-COOH and NIC-ER in PBS under continuous laser irradiation for 15 min. The remarkably high photostability of NIC-ER makes it very attractive for use in long-term tracking or fluorescence-guided surgery, which requires long periods of sustained light exposure [48].

**Fig. 3:**
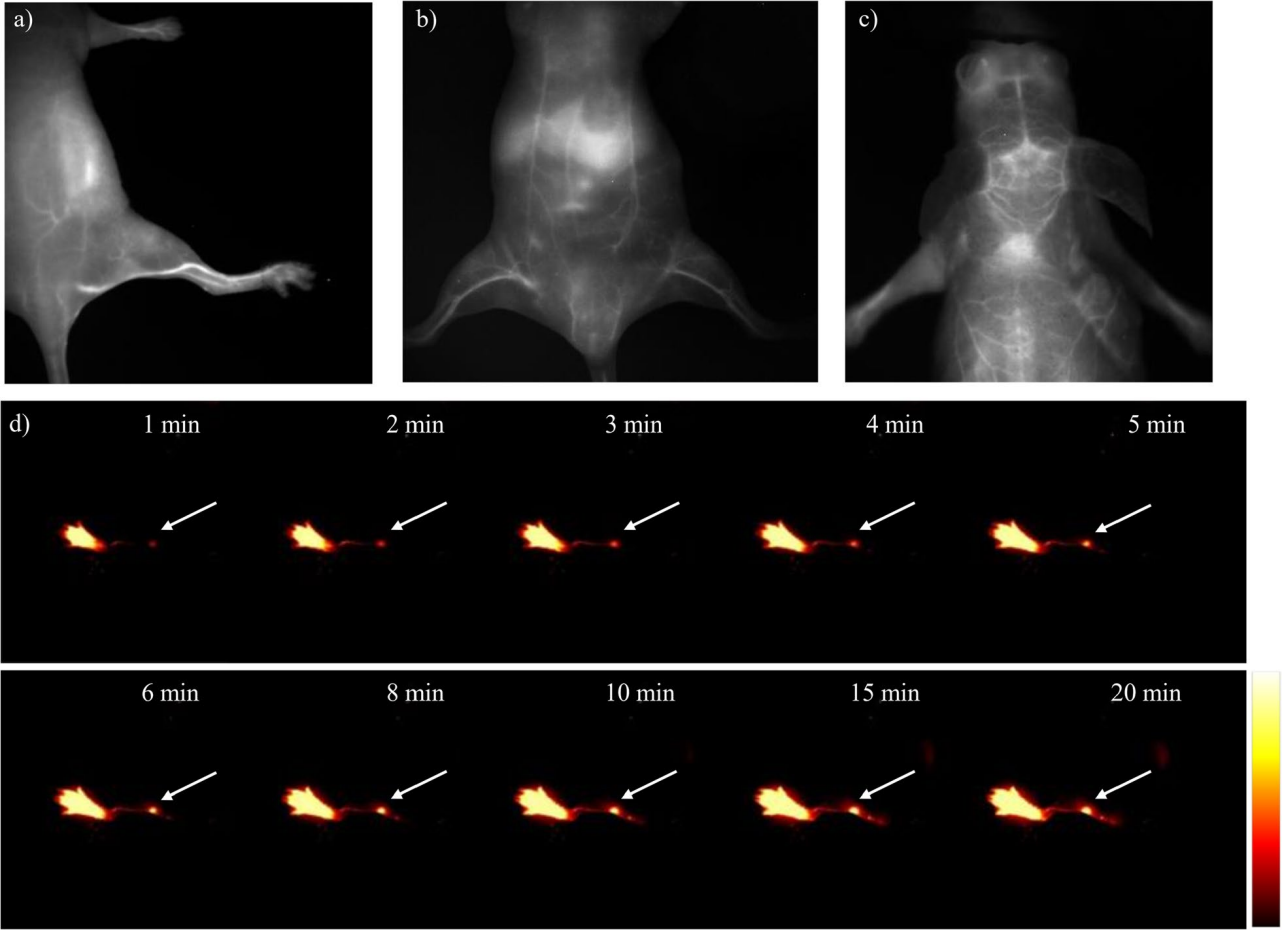
Fluorescence imaging of mouse vasculature in the NIR-II window (excitation = 915 nm, 1250 nm long-pass filter, 100 ms expose time). **a** NIR-II imaging of hind limb vasculature. **b** NIR-II imaging of abdominal vessels. **c** NIR-II imaging of mouse brain through intact scalp and skull vasculature. **d** Lymph node imaging after injection of NIC-ER in footpad, white arrows indicate the popliteal lymph node

We injected NIC-ER intravenously into BALB/c nude mice at the dose of 0.5 mg/Kg to investigate the potential of NIC-ER as a novel NIR-II contrast agent for bioimaging. Excitation was achieved using a 915 nm diode laser at 100 mW cm^−2^, and NIR-II fluorescence images were captured through a 1250 nm LP filter (exposure time, 100 ms). After intravenous injection of NIC-ER, the hind limb vessels were clearly distinguished, revealing the high spatial resolution of NIR-II fluorescent imaging (Fig. 3a). Meanwhile, the fluorescence intensities of blood vessels and muscles in the hind limb were measured, and the signal-to-background ratio (SBR) was evaluated to be 3.2 (Additional file 1: Figure S5). Furthermore, the vessel resolved by NIR-II showed a maximum feature resolution (full width at half maximum, FWHM) of 0.34 mm, which is consistent with the previously reported value [30]. Furthermore, the whole abdomen vessels, including the inferior epigastric, can be imaged (Fig. 3b). The above results inspired us to probe deep tissue. Compared with the skin surface vessels, cerebral vasculature is located much deeper (≈1.3 mm). We then further explored the imaging performance of NIC-ER for in vivo cerebral vasculature imaging through the scalp and the skull (cranial bone) without craniotomy. The large and detailed vessels were clearly distinguished (Fig. 3c) after intravenous injection of NIC-ER. Next, we explored the application of NIC-ER to lymphatic imaging. Fluorescence images were obtained after intradermal injection of NIC-ER at the rear paw of nude mice. Figure 3d shows that the popliteal lymph nodes and the lymphatic ducts were visualized very clearly. These results show that NIC-ER is qualified as a promising small-molecule organic dye with better imaging clarity and quality.

**Fig. 4:**
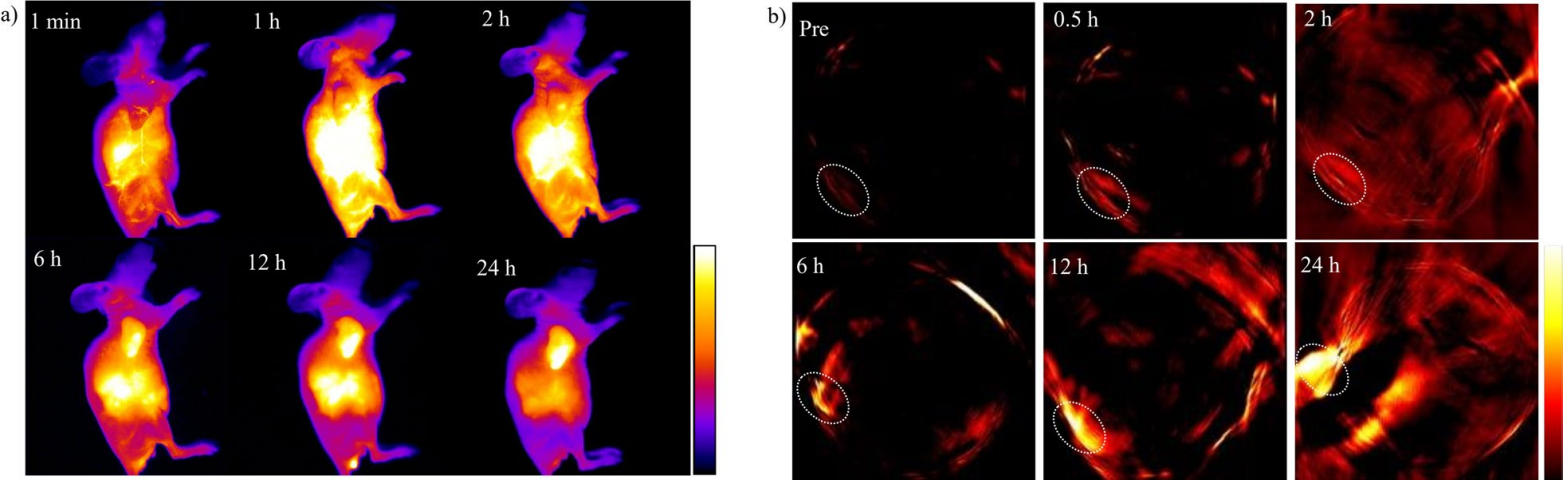
**a** The in vivo NIR-II images of the 4T1 tumor at different time points (1 min-24 h) after a tail vein injection of **NIC-ER**. **b** The in vivo PA images of the 4T1 tumor at different time points (pre-24 h) after a tail vein injection of **NIC-ER**, the dashed ellipses indicate the tumor

Inspired by the above excellent results, we further investigated the potential of NIC-ER for the precise detection of the tumor. The 4T1 tumor-bearing nude mice were injected with NIC-ER via tail vein. In vivo NIR-II imaging was performed at 1 min, 1, 2, 6, 12, and 24 h post-injection. The tumor-feeding blood vessels were unambiguously observed from the imaging data at 1 min. During the test time, the signal intensity in the tumor site gradually enhanced. The tumor could be clearly visualized from the surrounding background during 6–24 h post-injection (Fig. 4a). To further track the biodistribution of NIC-ER, the mice were sacrificed at 24 h post injection and their main organs were harvested for ex vivo imaging (Additional file 1: Figure S8). Strong NIR-II fluorescence signals at the liver, kidney and gut were observed, which suggested NIC-ER could be excreted from the body through hepatobiliary and renal system. The high tumor targeting efficacy of NIC-ER can be ascribed to the albumin-hitchhiking capability of NIC-ER in vivo. After IV injection, NIC-ER was spontaneously located into the pocket of albumin. Thus, the complex can take advantage of endogenous albumin transport pathways for tumor accumulation because tumor microenvironment overexpresses proteins that bind albumin, such as gp60 receptor and SPARC. Moreover, albumin is a macromolecule, and as such the EPR effect may also contribute to tumor uptake. Additionally, slow release of the NIC-ER from albumin over time would allow continual tumor uptake via RGD-mediated endocytosis. Collectively, the effective tumor uptake of NIC-ER can be ascribed to the result of a combination of RGD-mediated endocytosis, EPR effect and endogenous albumin transport pathways. This tumor targeting pattern in other tEB conjugates have been carefully investigated by Chen’s group.[32–36].

We also performed block study in in vivo NIR II imaging. Given the rapid clearance of RGD and slow pharmacokinetics of NIC-ER, the conjugates of maleimide-modified tEB and c (RGDfc) (termed as ER) was used in vivo to block the α_v_β_3_ integrin receptor. We can still observe gradually enhanced NIR II signal in tumor site, although the intensity was significantly lower than that in only NIC-ER group at all time points (Additional file 1: Figure S9). This result may indicate that the albumin binding NIC-ER can still accumulate in tumor site by EPR effect or endogenous albumin transport pathways although the α_v_β_3_ integrin receptor was blocked. Thus, the high tumor targeting efficacy of NIC-ER can largely be ascribed to the combination effect of RGD-mediated endocytosis, EPR effect and endogenous albumin transport pathways.

As mentioned above, NIC-ER exhibited strong absorption in the NIR region, making it have great potential as a PA imaging agent. Thus, the in vivo PA imaging was performed on a subcutaneous 4T1 breast cancer tumor-bearing mouse model. The PA tomography system recorded the PA images of tumor cross-sections at different time intervals. Figure 4b shows that a very weak PA signal can be observed under the excitation at 910 nm before intravenous injection of the NIC-ER, which may ascribe to the endogenous hemoglobin contrast in blood vessels. The PA signal of tumor tissue strengthened with the increase of time interval after the injection of NIC-ER, which is consistent with the in vivo NIR-II FL imaging behavior. These excellent imaging results demonstrate that NIC-ER offers an outstanding promise as a NIR-II and PA probe for the margin assessment of tissue localization and intraoperative surgery.

In cancer treatment, efficient therapy is always a challenging task [49]. Recently, photothermal therapy (PTT) has been proven to be a powerful therapeutic modality for cancer [50–53]. Thus, we investigated the possibility of NIC-ER for in vivo photothermal therapy. The temperature of NIC-ER in PBS or HSA solution was first monitored with the irradiation of the 808 nm or 915nm laser at 0.5 w/cm^2^. Figure S4 shows that the temperature of NIC-ER/HSA increased quickly in the first 3 min, while the rate decreased and reached a plateau within 5 min. The maximum temperature (T_max_) of NIC-ER/HSA reached 59 °C and was maintained at least for 10 min. However, free NIC-ER showed a slower temperature increment rate and lower maximum temperature (T_max_) when irradiated for the same time. Notably, the temperature of free NIC-ER was slightly decreased with the irradiation time above 13 min, which supports the previous observation of its slight instability under long-time light irradiation. Therefore, the stability and photothermal conversion ability of NIC-ER were improved after being bound to HSA, which would be helpful for cancer phototherapy. In order to visually evaluate the in vitro therapeutic effect of NIC-ER, the cells were stained with calcein-AM and propidium iodide (PI) to identify live and dead/late apoptotic cells, respectively. As shown in Additional file 1: Figure S10, cells all displayed green fluorescence in NIC-ER group, which suggested that NIC-ER alone cannot kill cells. On the contrary, most of cells death and exhibiting intense homogeneous red fluorescence in NIC-ER+Llaser group.

**Fig. 5:**
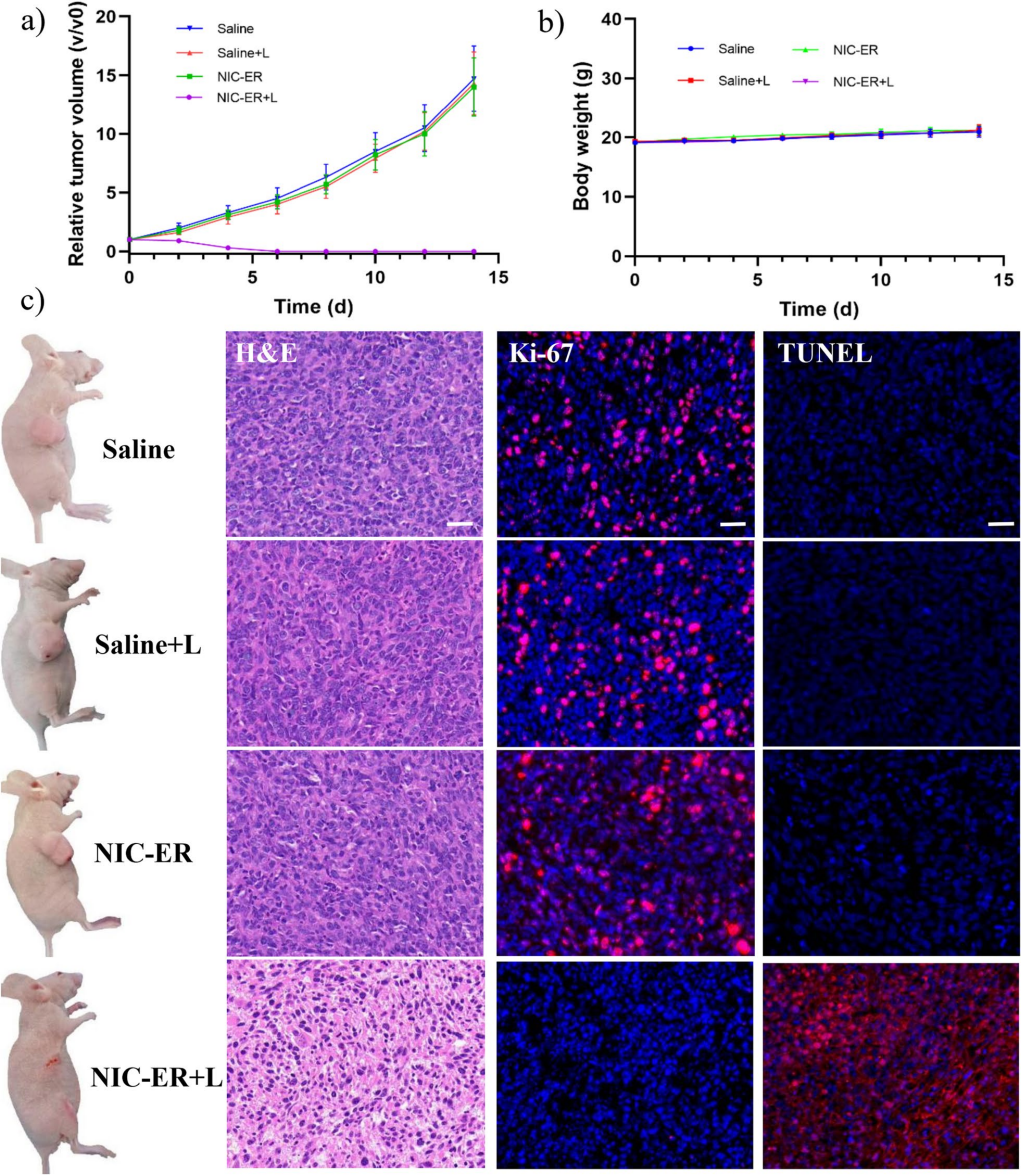
In vivo photothermal therapy study. **a** Growth curves of the xenografted 4T1 tumors on the mice (n = 3) after receiving treatment with saline alone, saline and laser irradiation, NIC-ER alone, and NIC-ER and laser irradiation. **b** Body-weight curves of the tumor-bearing mice after receiving the treatment indicated. **c** Representative images of tumor-bearing mice taken at 14 days post-treatment and Histological H&E, fluorescence TUNEL and Ki-67 staining of tumor slices post various treatments. Scale bar: 20 μm

The mice bearing 4T1 subcutaneous tumor were divided randomly into four groups (three mice in each group), which were named “saline”, “saline+laser”, “NIC-ER”, and “NIC-ER + laser”, respectively. For the PTT treatment group, the tumors of mice were continuously irradiated with a 915 nm laser for 5 min at 12 h after intravenous injection of NIC-ER. The in vivo antitumor efficacies of the treatments mentioned above were examined by monitoring the tumor volumes for 14 days (the treatments were performed on day 0). The tumors treated with saline, laser, and NIC-ER without laser irradiation grew rapidly, indicating that the 4T1 tumor growth werenot affected by saline, NIC-ER, or laser alone irradiation. In contrast, the “NIC-ER + laser” treatment via PTT exhibits an excellent antitumor efficacy, the growth of the 4T1 tumor was completely inhibited and did not recur during the experiment time (Fig. 5a). Notably, the excellent photothermal activity and the prominent tumor uptake ability of NIC-ER affords such highly effective tumor growth suppression. In addition, there is no significant body weight change in mice in these four groups, suggesting that photothermal treatment was rationally well-tolerated (Fig. 5b).

 Furthermore, we studied the therapeutic effect of these four groups at the microscopic level (Fig. 5c). In this experiment, the mice in all four groups were sacrificed, and the tumors were excised for hematoxylin and eosin (H&E) staining and immune-histochemical analyses, including terminal deoxynucleotidyl transferase dUTP nick end labeling (TUNEL) and transcription factors Ki-67 (Ki-67). Figure 5c shows that tumors in control groups (saline, saline + laser, and NIC-ER) had tightly packed cancer cells with normal morphology, and no necrosis or obvious apoptosis was observed. In contrast, the obvious nuclear reduction was observed in the “NIC-ER + laser” treatment group. The TUNEL staining results showed that the apoptotic cells of tumor tissues had significantly increased in “NIC-ER + laser” treatment group, but not in the other three groups. Ki-67 staining results indicated that the proliferation capacity of tumor tissue was obviously reduced in “NIC-ER + laser” treatment group as compared to those in the other three groups. Both TUNEL and Ki-67 staining confirm that “NIC-ER + laser” treatment is the most efficacious in inducing apoptosis and suppressing the proliferation capacity of tumor cells. These results proved the great potential of NIC-ER as a highly effective therapeutic agent.

## Conclusions

In summary, this study provides a delicate molecular design strategy for developing superior NIR-II cyanine dye with sufficient brightness, large Stokes-shift, and high photostability. Compared to the symmetrical cyanine dye ICG, NIC has a nonsymmetrical structure by replacing only indoline donors with a fully conjugated benz[c,d]indole donor. This minor structure alternation affords NIC with NIR-II emission and high extinction coefficient. NIC-ER with endogenous albumin-hitchhiking capability was constructed to further enhance its in vivo fluorescence brightness. In the presence of albumin, NIC-ER was spontaneously located into the pocket of albumin, and a brilliant ~89-fold increase in fluorescence was observed. NIC-ER in HSA exhibits bright NIR-II emission with high photostability and significant Stokes shift (>110 nm) due to the high molar absorptivity and moderate quantum yield. NIC-ER exhibits NIR-II/PA bimodal imaging capabilities and favorable photothermal effect. Thus, noninvasive NIR-II imaging of small blood vessels at limb, abdomen, and cerebral regions was achieved with a high signal-to-background ratio and deep penetration. NIC-ER has also been used for targeted NIR-II fluorescence and NIR-I PA imaging of subcutaneous tumors. Moreover, NIC-ER achieved efficient photothermal elimination of tumors without recurrence due to its excellent photothermal conversion capability and tumor-targeting ability. The above results proved that NIC-ER has great potential in clinical applications for tumor imaging and photothermal therapy. Overall, our strategy may open a new avenue for designing and constructing high-performance NIR-II fluorophores.

## Supplementary Information

**Additional file 1: Figure S1.** Cell viability of Hela cells after incubated with NIC-ER with various concentrations for 48 h. **Figure S2.** Absorbance intensity change of NIC-ER in HSA/PBS/Blood and ICG-COOH in PBS after repeated laser irradiation. NIR-II fluorescence image of NIC-ER in HSA or Blood. **Figure S3.** Photo of ICG-COOH (left) and NIC (right) in DMSO. **Figure S4.** Temperature curves of NIC-ER in HSA or in PBS and HSA alone under NIR laser irradiation. **Figure S5.** NIR-II imaging of hind limb vasculature with the vessel FWHM width (white line) analysis. Scale bar: 5 mm. **Figure S6.** NIR-II imaging of cerebral vasculature with the vessel FWHM width (white line) analysis. Scale bar: 5 mm. **Figure S7.** Histological H&E staining for main organs (heart, liver, spleen, lung and kidney) of the mice intravenously administrated with PBS and NIC-ER (10 times than the imaging doses, 5 mg of NIC-ER per kg mouse) for 7 days. Scale bar: 50 μm. **Figure S8.** Ex vivo images of main organs of NIC-ER treated mice. **Figure S9.** The in vivo NIR-II images of the 4T1 tumor at different time points (1–24 h) after co-injection of NIC-ER and ER (the conjugates of EB and cRGD, 0.4 mg/mouse). **Figure S10.** Calcein-AM/propidium iodide (PI) staining of 4T1 cells pretreated with NIC-ER with or without laser for 5 min at 0.5 W/cm^2^. **Table S1.** Overview of the names and chemical structures of key compounds. **Table S2.** The absolute quantum yield of NIC-ER in DMSO, PBS and HSA (3 mg/mL). **Scheme S1.** The synthetic route of NIC-ER. **Figure S11.** 1H NMR spectroscopy of compound of NIC. **Figure S12.** 13C NMR spectroscopy of compound of NIC. **Figure S13.** MS spectroscopy of NIC. **Figure S14.** MALDI-TOF-MS measurement of NIC-MLEB. **Figure S15.** MALDI-TOF-MS measurement of NIC-ER.

## Data Availability

All data generated or analysed during this study are included in this published article.
