# Supplementary material for: Unsymmetrical cyanine dye via in vivo hitchhiking endogenous albumin affords high-performance NIR-II/photoacoustic imaging and photothermal therapy
Source: J Nanobiotechnology. 2021 Oct 24;19:334. doi: 10.1186/s12951-021-01075-0 (PMC8543934; doi:10.1186/s12951-021-01075-0)
Supplement: Supplementary file 1 — Additional file 1: Figure S1. Cell viability of Hela cells after incubated with NIC-ER with various concentrations for 48 h. Figure S2. Absorbance intensity change of NIC-ER in HSA/PBS/Blood and ICG-COOH in PBS after repeated laser irradiation. NIR-II fluorescence image of NIC-ER in HSA or Blood. Figure S3. Photo of ICG-COOH (left) and NIC (right) in DMSO. Figure S4. Temperature curves of NIC-ER in HSA or in PBS and HSA alone under NIR laser irradiation. Figure S5. NIR-II imaging of hind limb vasculature with the vessel FWHM width (white line) analysis. Scale bar: 5 mm. Figure S6. NIR-II imaging of cerebral vasculature with the vessel FWHM width (white line) analysis. Scale bar: 5 mm. Figure S7. Histological H&E staining for main organs (heart, liver, spleen, lung and kidney) of the mice intravenously administrated with PBS and NIC-ER (10 times than the imaging doses, 5 mg of NIC-ER per kg mouse) for 7 days. Scale bar: 50 μm. Figure S8. Ex vivo images of main organs of NIC-ER treated mice. Figure S9. The in vivo NIR-II images of the 4T1 tumor at different time points (1–24 h) after co-injection of NIC-ER and ER (the conjugates of EB and cRGD, 0.4 mg/mouse). Figure S10. Calcein-AM/propidium iodide (PI) staining of 4T1 cells pretreated with NIC-ER with or without laser for 5 min at 0.5 W/cm2. Table S1. Overview of the names and chemical structures of key compounds. Table S2. The absolute quantum yield of NIC-ER in DMSO, PBS and HSA (3 mg/mL). Scheme S1. The synthetic route of NIC-ER. Figure S11. 1H NMR spectroscopy of compound of NIC. Figure S12. 13C NMR spectroscopy of compound of NIC. Figure S13. MS spectroscopy of NIC. Figure S14. MALDI-TOF-MS measurement of NIC-MLEB. Figure S15. MALDI-TOF-MS measurement of NIC-ER. [file 12951_2021_1075_MOESM1_ESM.docx]

**Additional file 1 for**

**Unsymmetrical cyanine dye via** ***in vivo* hitchhiking endogenous albumin affords high-performance NIR-II/photoacoustic imaging and photothermal therapy.**

Pengfei Xu ^a,b^, Linan Hu^c^, Cheng Yu^c^, Fei Kang^a^, Weidong Yang^a^, Mingru Zhang^a^, Pei Jiang^b^* and Jing Wang ^a^*

^a^Department of Nuclear Medicine, Xijing Hospital, Fourth Military Medical University. 710032. #127 West Changle Road, Xi'an, Shaanxi, China;

^b^Institute of Clinical Pharmacy & Pharmacology, Jining First People’s Hospital, Jining Medical University, Jining 272000, P.R. China.

^c^Departments of Radiology, The Second Xiangya Hospital, Central South University, Changsha, Hunan 410011, P.R. China.

* Author of correspondence:

Dr. Pei Jiang, Institute of Clinical Pharmacy & Pharmacology, Jining First People’s Hospital, Jining Medical University, Jining 272000, P.R. China. Email: jiangpeicsu@sina.com.

Prof. Jing Wang, Department of Nuclear Medicine, Xijing Hospital, Fourth Military Medical University. 710032. #127 West Changle Road, Xi'an, Shaanxi, China. Email: wangjing@fmmu.edu.cn

**Materials and characterization**

All chemical reagents were obtained from the commercial suppliers and used without further purification. The ^1^H and ^13^C NMR spectra were acquired on Bruker 400 or 600 MHz magnetic resonance spectrometers. Data for ^1^H NMR spectra are reported as follows: chemical shifts are reported as δ in units of parts per million (ppm) relative to chloroform-d (δ 7.26, s); coupling constants are reported as a *J* value in Hertz (Hz); multiplicities are reported as follows: s (singlet), d (doublet), t (triplet), q (quartet), dd (doublet of doublets) and m (multiplet); the number of protons (n) for a given resonance is indicated nH based on the spectral integration values. MALDITOF-MS spectra were obtained by an AB SCIEX 4700 TOF/TOF System. The nanoparticle morphology was studied by field emission transmission electron microscopy (FE-TEM). UV-vis was tested with a Shimadzu Model UV-1700 spectrometer. FSL1000 fluorescence spectrophotometer (FSL1000, Edinburgh Instruments Ltd.) equipped with the Quantum Yield measuring accessory and Report Generator program was used for photoluminescence (PL) spectra study and Absolute Quantum Yield Measurement. Flash chromatography was performed on 200-400 mesh silica. Thin layer chromatography was performed using silica gel 60G F254 25 glass plates and visualized under 254/365 nm ultraviolet light. Density functional theory (DFT) calculations were conducted by the Gaussian 09 program. NIR-II in vivo imaging was performed on a small animal imaging system with fiber-coupled 915 nm laser system.

Docking modeling

The crystal structure of human serum albumin (HSA, PDB entry: 4K2C) was retrieved from Protein Data Bank (http://www.pdb.org/pdb/) and then prepared by Discovery Studio 2.5 (Discovery Studio, version 2.5, 2009, Accelrys Inc., San Diego, CA, USA.), including residues repair and energy minimization. We used CDocker program in Discovery Studio 2.5 to construct the 3D models of HSA and **NIC-ER**. The dock program CDocker and DS catalyst Score are applied to construct receptoreligand complexes. The binding site of the receptor is set at the active site of HSA with a radius of 5 Å, large enough to cover the binding pocket. CDocker is a grid-based molecular docking method that employs CHARMM forcefield. The receptor is held rigid while the ligands are allowed to flex during the refinement. For predocked ligands, prior knowledge of the binding site is not required. It is possible, however, to specify the ligand placement in the active site using a binding site sphere. Random ligand conformations are generated from the initial ligand structure through high temperature molecular dynamics, followed by random rotations. The random conformations are refined by grid-based simulated annealing and a final grid-based or full forcefield minimization.

Density functional theory calculations.

All the calculations were performed in the gas phase using a Gaussian 09 program. The ground-state structure was optimized with B_3_LYP method and 6-311G(d, p) basis set. Then, a vertical excitation was carried out based on the optimized structure with the same method, from which the ground-state molecular orbital energy was obtained.

**Cell line and animal model.**

4T1 and Hela cells were obtained from the Type Culture Collection of the Chinese Academy of Sciences and culture media was obtained from Invitrogen Co. (Carlsbad, CA, USA). The cells were cultured in Dulbecco's modified Eagle’s medium (DMEM) supplemented with 10% (v/v) fetal bovine serum and 1% (v/v) penicillin at 37°C and 5% CO_2_. The 4T1 tumor model were established by subcutaneous injection of 4T1 (~1 × 10^6^ in 100 μL of PBS) into the front flank of female athymic nude mice and tumors developed within four weeks.

***In Vitro* Toxicity Study.**

To evaluate **NIC-ER**’s cytotoxicity, HeLa cells with a certain concentration (5 ×10^4^ cells in 100 μL medium per well) were carefully seeded in fresh 96-well plates. **NIC-ER** of changing concentrations were added into the cells. After further culture for 48h, CCK-8 assay was conducted to evaluate the relative cell viability.


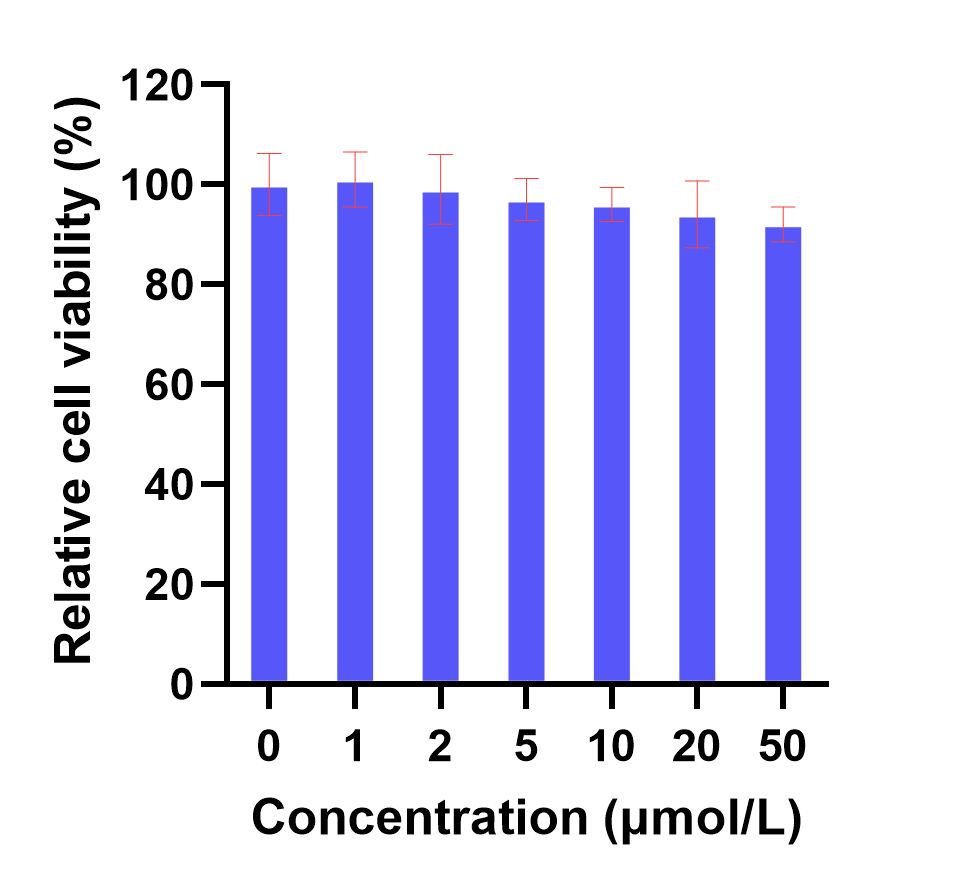


**Figure S1.** Cell viability of Hela cells after incubated with **NIC-ER** with various concentrations for 48 h.

***In Vivo* NIR-II fluorescence and PA imaging.**

Before conducting experiments, 2% isoflurane in oxygen was used to anesthetize mice in prone position. Three mice were used during parallel experiments to collect all the in vivo data. To study the imaging ability, mice were imaged before dot injection to record the background signal. After mice were intravenously injected with **NIC-ER** (0.5 mg/Kg), the NIR-II fluorescence imaging with thermoelectric cooled InGaAs camera was used to collect images over different time points, respectively. 915 nm continuous irradiation (100 mW/cm^2^) was used as the light source in the NIR-II fluorescence imaging system with exposure time of 100 ms equipped with 1250 nm long-pass filter (1250 LP). The signal/background ratio was processed using Image J software by counting six points and obtaining the average value. PA images and corresponding PA intensities at 910 nm were obtained with a PA microscopy system (Vevo LAZR, VisualSonics).

***In vivo* photothermal therapy**

When the tumor sizes reached about 100 mm^3^ after inoculation of 4T1 cells, the mice were parted into four groups (n = 3 per group) for different treatments: PBS alone; PBS plus NIR laser at 0.5 W/cm^2^ for 5 min (PBS + L); **NIC-ER** alone (**NIC-ER**); **NIC-ER** with NIR laser at 0.5 W/cm^2^ for 5 min (**NIC-ER** + L). After intravenous injection and NIR irradiation, the tumor sizes (length and width) and body weights were acquired with an electronic vernier caliper. Tumor sizes were calculated by this formula: tumor volume = A × B^2^ × 0.5. In this formula, A and B represents the length and width of tumors, respectively.

Histological Studies.

All the mice were sacrificed after complete treatment. The tumor were excised, fixed in 4% formalin solution, and sectioned at 5 μm thickness. After conventional H&E staining, the slices were examined with a digital microscope (Leica QWin). The fluorescent transcription factors Ki-67 (Ki-67) staining was conducted following common immunohistochemical steps. The fluorescent terminal deoxynucleotidyl transferase dUTP nick end labeling (TUNEL) staining was conducted following manual instruction of DeadEnd fluorometric TUNEL system kit (Promega, USA). The nuclei were counterstained with 4',6-diamidino-2-phenylindole (DAPI) containing a mounting solution (Dapi-Fluoromount-G, Southern Biotech, England).

**Photostability study.**

**NIC-ER** in 25 mg/mL HSA or in blood was exposed with a laser (915 nm, 0.5 W/cm^2^, 15 min) while the cyanine dye ICG-COOH and **NIC-ER** in PBS were exposed with a laser (808 nm, 0.5 W/cm^2^, 15 min). During these measurements, the absorptions of above solutions were monitored.


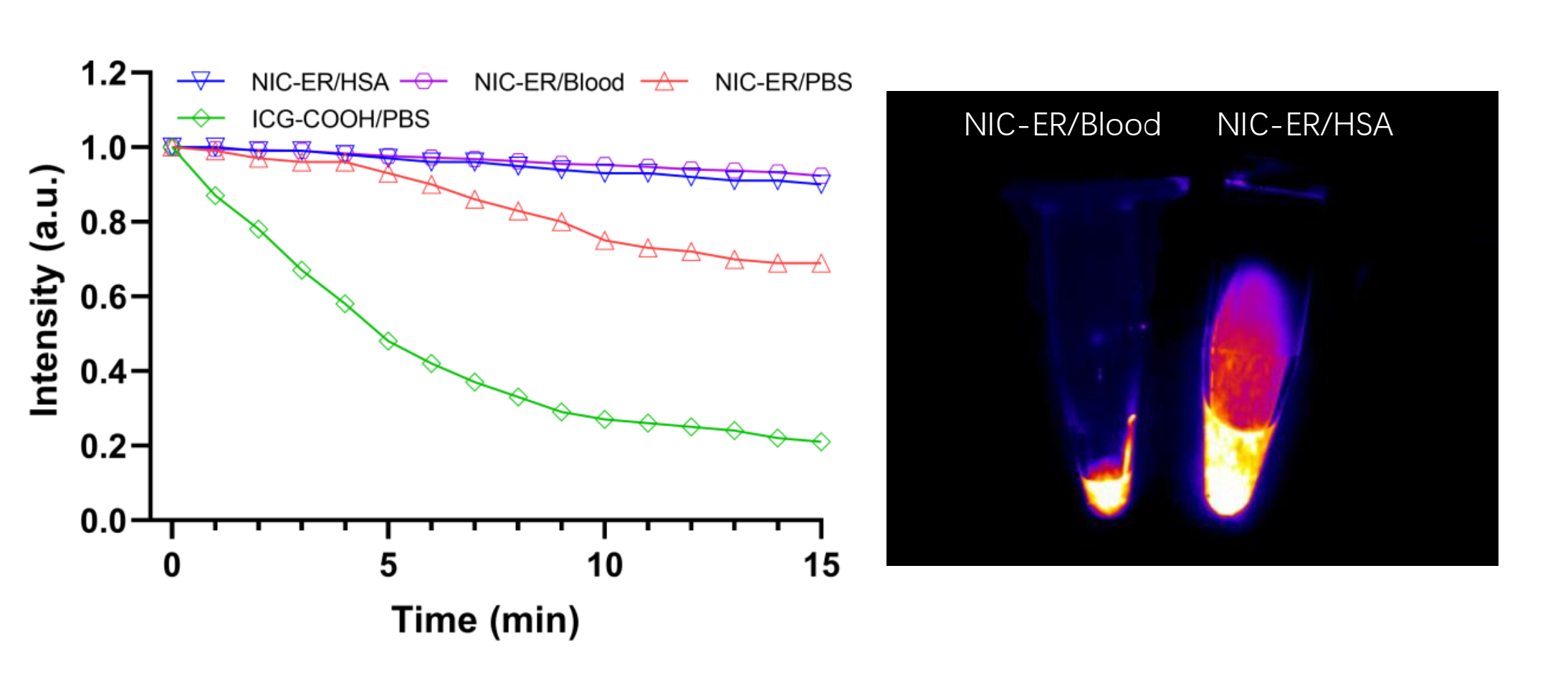


**Figure S2**. Absorbance intensity change of NIC-ER in HSA/PBS/Blood and ICG-COOH in PBS after repeated laser irradiation. NIR-II fluorescence image of NIC-ER in HSA or Blood.


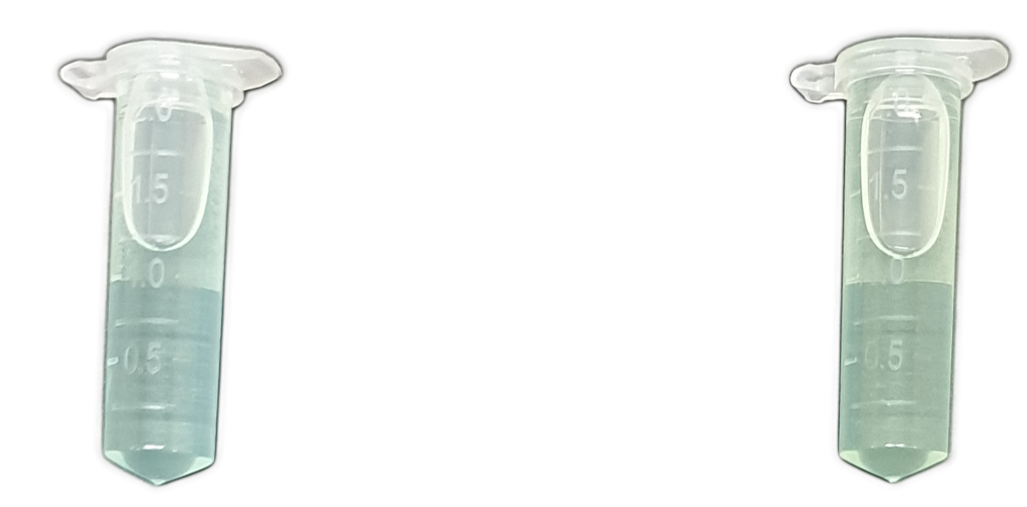


**Figure S3.** Photo of ICG-COOH (left) and NIC (right) in DMSO.

**
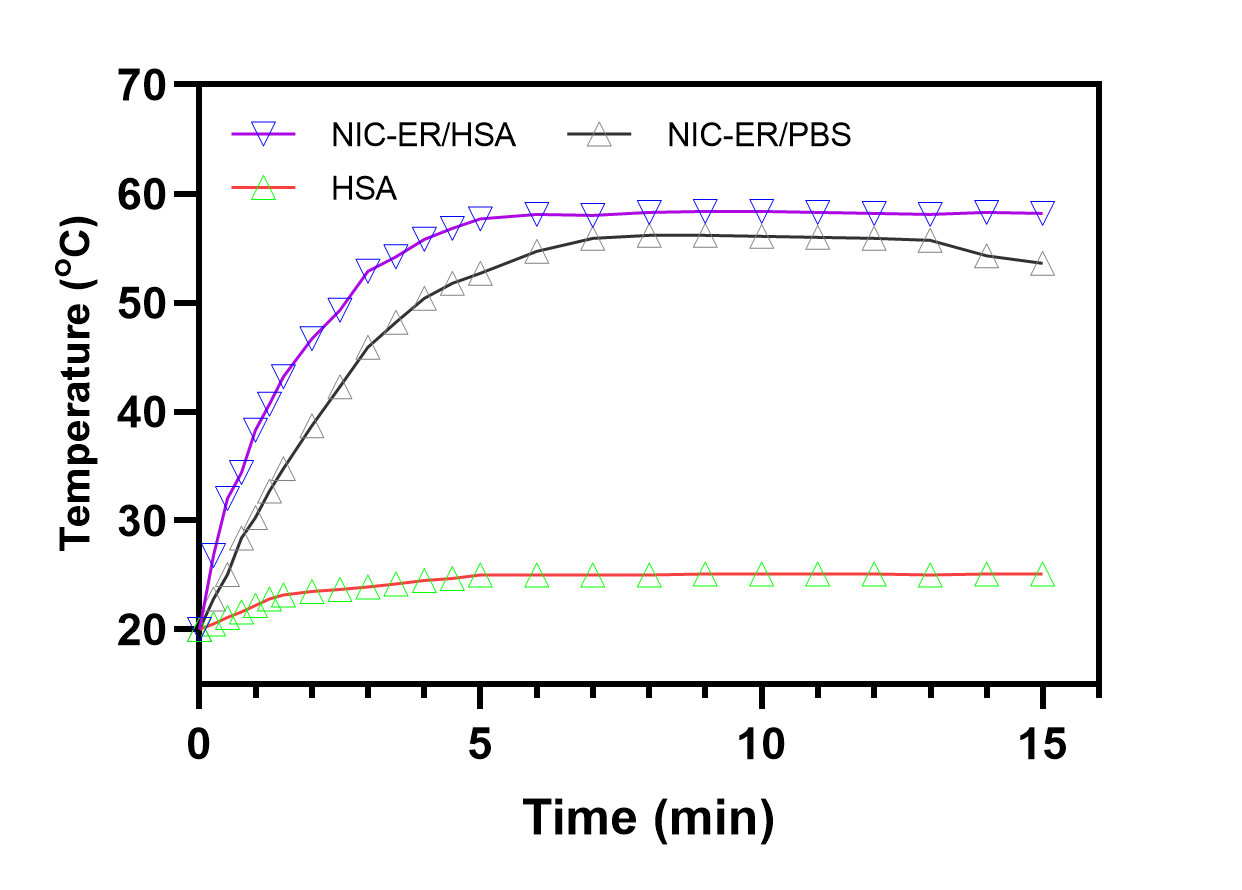
**

**Figure S4.** Temperature curves of NIC-ER in HSA or in PBS and HSA alone under NIR laser irradiation.

**
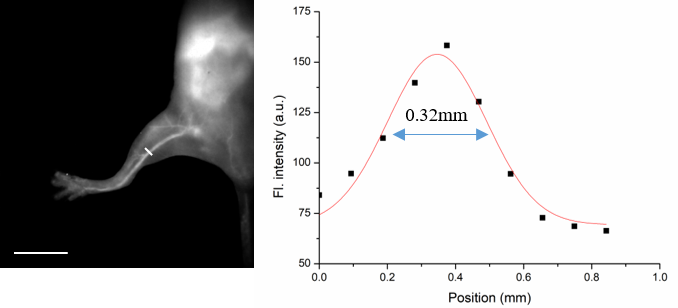
**

**Figure S5.** NIR-II imaging of hind limb vasculature with the vessel FWHM width (white line) analysis. Scale bar: 5 mm


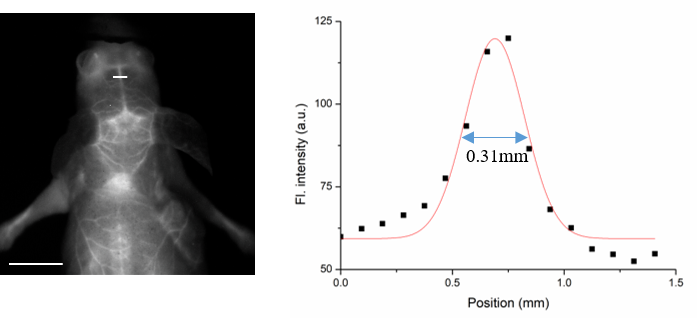


**Figure S6.** NIR-II imaging of cerebral vasculature with the vessel FWHM width (white line) analysis. Scale bar: 5 mm


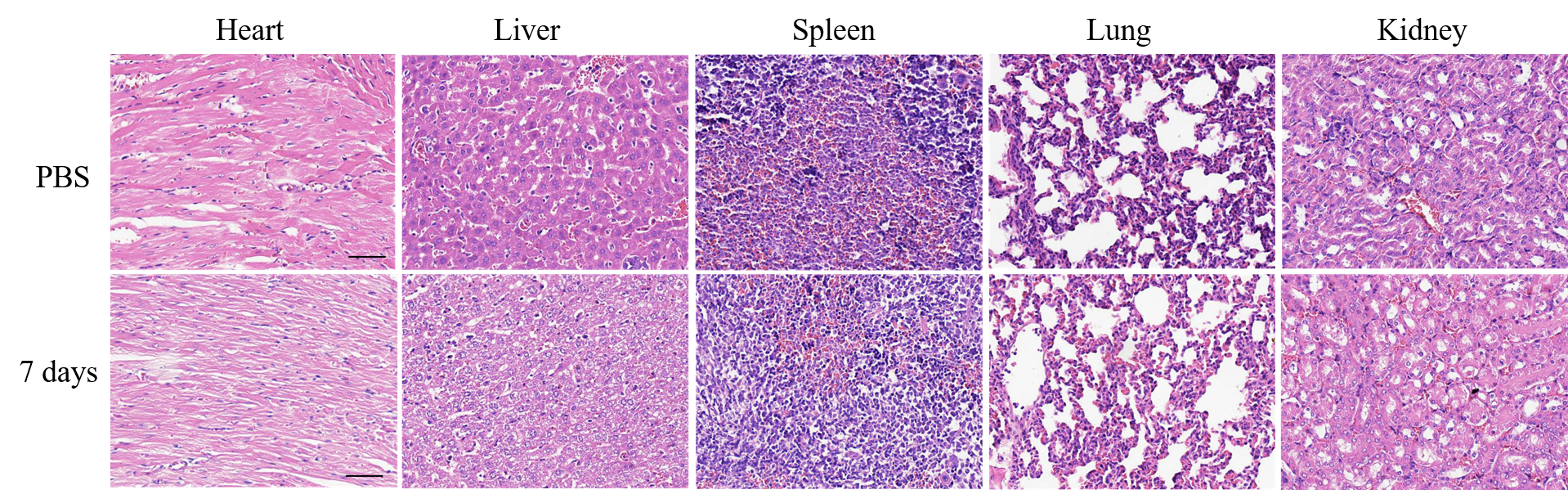


**Figure S7**. Histological H&E staining for main organs (heart, liver, spleen, lung and kidney) of the mice intravenously administrated with PBS and **NIC-ER** (10 times than the imaging doses, 5 mg of **NIC-ER** per kg mouse) for 7 days. Scale bar: 50 μm.


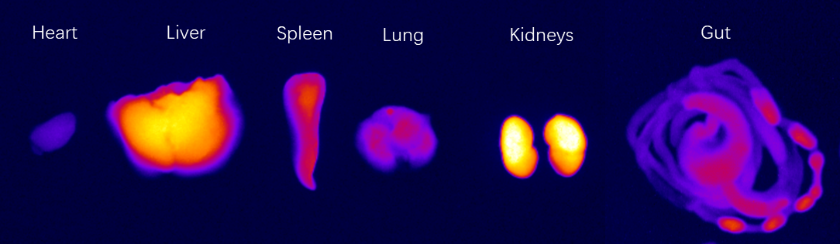


**Figure S8**. Ex vivo images of main organs of NIC-ER treated mice.


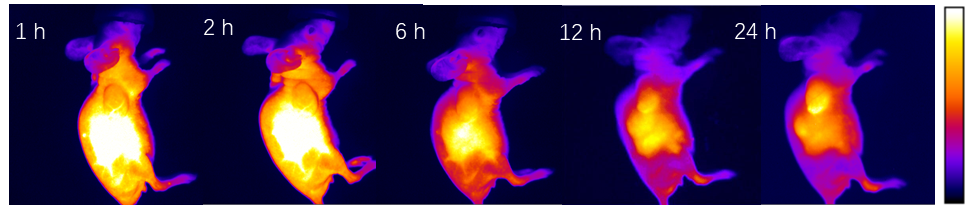


**Figure S9**. The *in vivo* NIR-II images of the 4T1 tumor at different time points (1h-24h) after co-injection of **NIC-ER** and ER (the conjugates of EB and cRGD, 0.4 mg/mouse).


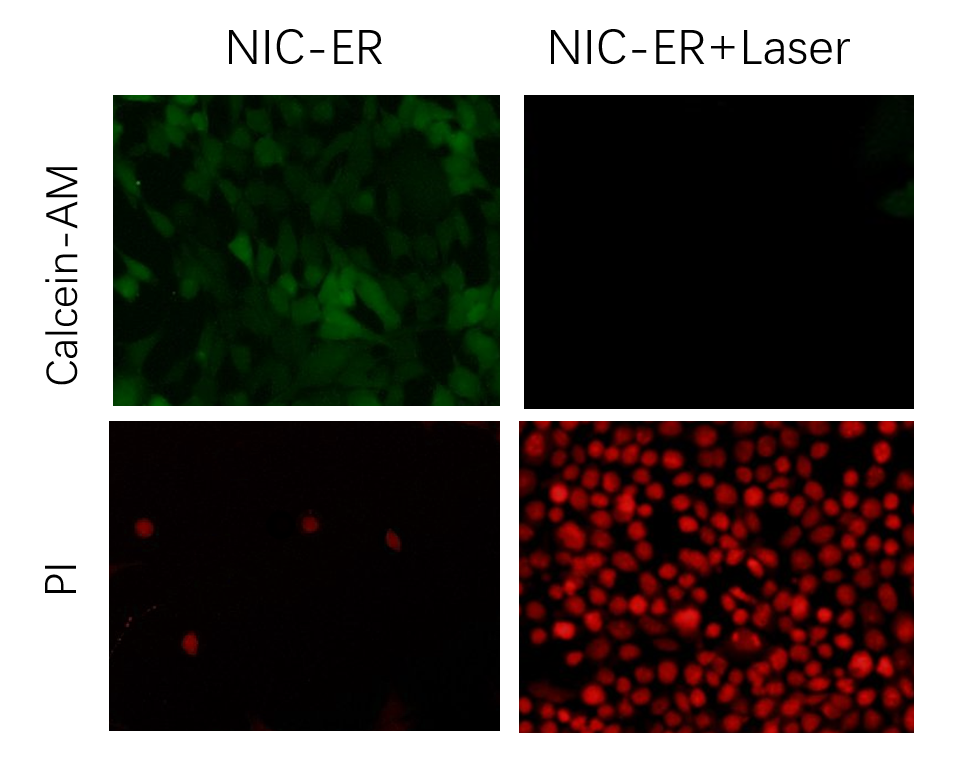


**Figure S10**. Calcein-AM/propidium iodide (PI) staining of 4T1cells pretreated with NIC-ER with or without laser for 5 min at 0.5 W/cm^2^.

**Table S1**. Overview of the names and chemical structures of key compounds.

| ICG-COOH | 4-((E)-2-((2E,4E,6E)-7-(3-(5-carboxypentyl)-1,1-dimethyl-1H-benzo[e]indol-3-ium-2-yl)hepta-2,4,6-trien-1-ylidene)-1,1-dimethyl-1H-benzo[e]indol-3(2H)-yl)butane-1-sulfonate |  |
| --- | --- | --- |
| ICG | 4-((E)-2-((2E,4E,6E)-7-(1,1-dimethyl-3-(4-sulfobutyl)-1H-benzo[e]indol-3-ium-2-yl)hepta-2,4,6-trien-1-ylidene)-1,1-dimethyl-1H-benzo[e]indol-3(2H)-yl)butane-1-sulfonate |  |
| C(RGDfc) | 2-((2S,5R,8R,11S)-5-benzyl-11-(3-guanidinopropyl)-8-(mercaptomethyl)-3,6,9,12,15-pentaoxo-1,4,7,10,13-pentaazacyclopentadecan-2-yl)acetic acid |  |
| MLEB | (E)-4-amino-6-((4'-(6-amino-2-(3-(2,5-dioxo-2,5-dihydro-1H-pyrrol-1-yl)propanamido)hexanamido)-3,3'-dimethyl-[1,1'-biphenyl]-4-yl)diazenyl)-5-hydroxynaphthalene-1,3-disulfonic acid |  |
| truncated Evans Blue(tEB) | (E)-4-amino-6-((4'-amino-3,3'-dimethyl-[1,1'-biphenyl]-4-yl)diazenyl)-5-hydroxynaphthalene-1,3-disulfonic acid |  |
| Evans Blue(EB) | 6,6'-((1E,1'E)-(3,3'-dimethyl-[1,1'-biphenyl]-4,4'-diyl)bis(diazene-2,1-diyl))bis(4-amino-5-hydroxynaphthalene-1,3-disulfonic acid) |  |
| ER | 2-((2S,5R,8R,11S)-8-(((1-(3-((4'-((E)-(8-amino-1-hydroxy-5,7-disulfonaphthalen-2-yl)diazenyl)-3,3'-dimethyl-[1,1'-biphenyl]-4-yl)amino)-3-oxopropyl)-2,5-dioxopyrrolidin-3-yl)thio)methyl)-5-benzyl-11-(3-guanidinopropyl)-3,6,9,12,15-pentaoxo-1,4,7,10,13-pentaazacyclopentadecan-2-yl)acetic acid |  |

**Table S2**. The absolute quantum yield of NIC-ER in DMSO, PBS and HSA (3 mg/mL).

| Solvent | QYs |
| --- | --- |
| DMSO | 1.39% |
| PBS | 0.12% |
| HSA (3 mg/mL) | 1.23% |

**Synthesis details**

**Scheme S1**. The synthetic route of **NIC-ER**.

Synthesis and Characterization of **NIC**. The compound 1 (690 mg, 2.0 mmol), *N*-[5-(phenylamino)-2,4-pentadienylidene]aniline monohydrochloride (654 mg, 2.0 mmol) and AcONa (328 mg, 4.0mmol) were added to Ac_2_O (10 mL) and the mixture was stirred at 80 ^o^C for 2 h under vigorous stirring with a maintained positive pressure of nitrogen. After the solution cool down to room temperature, water (30 mL) was added to the mixture, followed by extraction with CH_2_Cl_2_ (15 mL x 3). The combined organic phase was dried by anhydrous Na_2_SO_4_. The solvent was removed by evaporation, and Ac_2_O (5 mL), pyridine (5 mL) and compound 2 (720 mg, 2.0 mmol) was added. The resulting mixture was stirred for 10 min, and then allowed to warm gradually to 50 ^o^C in an oil bath. The reaction mixture was further stirred for 10 h at this temperature, and then cooled to room temperature. The solvent was removed by evaporation and the residues was purified by flash column chromatographyon silica gel to afford pure sample (799 mg, 58%) as a solid. ^1^H NMR (600 MHz, DMSO) δ 12.02 (s, 1H), 8.36 (dd, *J* = 22.9, 7.1 Hz, 2H), 8.17 (t, *J* = 11.8 Hz, 2H), 8.12 (d, *J* = 8.1 Hz, 1H), 8.07 (d, *J* = 8.1 Hz, 1H), 7.98 (d, *J* = 8.3 Hz, 2H), 7.85 (t, *J* = 12.1 Hz, 1H), 7.74 (dt, *J* = 19.8, 7.6 Hz, 2H), 7.63 – 7.59 (m, 1H), 7.53 – 7.49 (m, 1H), 7.46 (d, *J* = 8.0 Hz, 1H), 7.12 (d, *J* = 6.6 Hz, 1H), 7.02 (d, *J* = 13.8 Hz, 1H), 6.81 (d, *J* = 12.1 Hz, 2H), 6.56 (d, *J* = 12.1 Hz, 1H), 4.46 (s, 2H), 4.08 (s, 2H), 2.59 (t, *J* = 6.3 Hz, 2H), 2.22 (t, *J* = 7.2 Hz, 2H), 1.93 (d, *J* = 16.2 Hz, 8H), 1.85 (d, *J* = 6.3 Hz, 2H), 1.76 – 1.69 (m, 2H), 1.62 – 1.53 (m, 2H), 1.43 (d, *J* = 6.8 Hz, 2H). ^13^C NMR (151 MHz, DMSO) δ 177.99, 174.84, 153.73, 153.35, 139.51, 136.45, 132.72, 131.20, 130.56, 130.43, 129.93, 129.86, 129.80, 128.50, 128.45, 127.60, 126.51, 125.92, 123.17, 119.10, 113.04, 109.93, 105.43, 52.42, 51.04, 45.58, 34.05, 28.27, 27.28, 26.75, 26.36, 24.74, 22.83. ESI-MS: [Calcd. for C_42_H_45_N_2_O_5_S^+^: 689.30, found: m/z 689.50.]

Synthesis and Characterization of **NIC-MLEB**. **NIC** (13.7 mg,0.02 mmol) in 5 mL anhydrous N, N-dimethylformamide (DMF) was stirred with compound **MLEB** (16.4 mg, 0.02 mmol) with the addition of DIPEA (8 mg, 0.06 mmol). Stirred for 2 mins and then added HBTU 5mg (7.5 mg, 0.02 mmol). The reaction solution was stirred over night at room temperature under a nitrogen atmosphere. After the reaction finished, 500 µL of water was added and stirred for 1h to quench the excess HBTU. The reaction mixture was monitored by analytical HPLC, which was performed using Symmetry C-18 columns from YMC (3 μm, 150 × 4.6 mm i.d.). The mobile phases (A) demineralized water and (B) acetonitrile were acidified to pH 3 with trifluoroacetic acid. Gradient elution was performed as follows: 10% of B, 0-3 min;10-90% of B, 3-15 min; 90% of B, 15-16 min; 90-10% of B, 16-18min; 10% of B, 18-20min. The product was purified by Pro-HPLC. Lyophilization of the purified material gave 12.5 mg (42 %) of **NIC-ER**. MALDI-TOF [Calcd. for C_79_H_82_N_9_O_15_S_3_^+^: 1492.51, found: m/z 1492.158.]

Synthesis and Characterization of **NIC-ER**. **NIC-MLEB** (7.5 mg, 5 μmol) was dissolved in 0.5 mL of DMSO. RGD-SH (3.9 mg, 5 μmol) was dissolved in 0.5 mL of de-gassed 0.1% sodium ascorbate (w/v) in phosphate buffer-saline (PBS) and added to the reaction solution. The reaction was stirred at RT for 2 h. The reaction mixture was monitored by analytical HPLC, which was performed using Symmetry C-18 columns from YMC (3 μm, 150 × 4.6 mm i.d.). The mobile phases (A) demineralized water and (B) acetonitrile were acidified to pH 3 with trifluoroacetic acid. Gradient elution was performed as follows: 10% of B, 0-3 min;10-90% of B, 3-15 min; 90% of B, 15-16 min; 90-10% of B, 16-18min; 10% of B, 18-20min. The product was purified by Pro-HPLC. Lyophilization of the purified material gave 5.58 mg (54%) of **NIC-ER**. MALDI-TOF [Calcd. for C_103_H_116_N_17_O_22_S_4_^+^: 2070.74, found: m/z 2070.758].


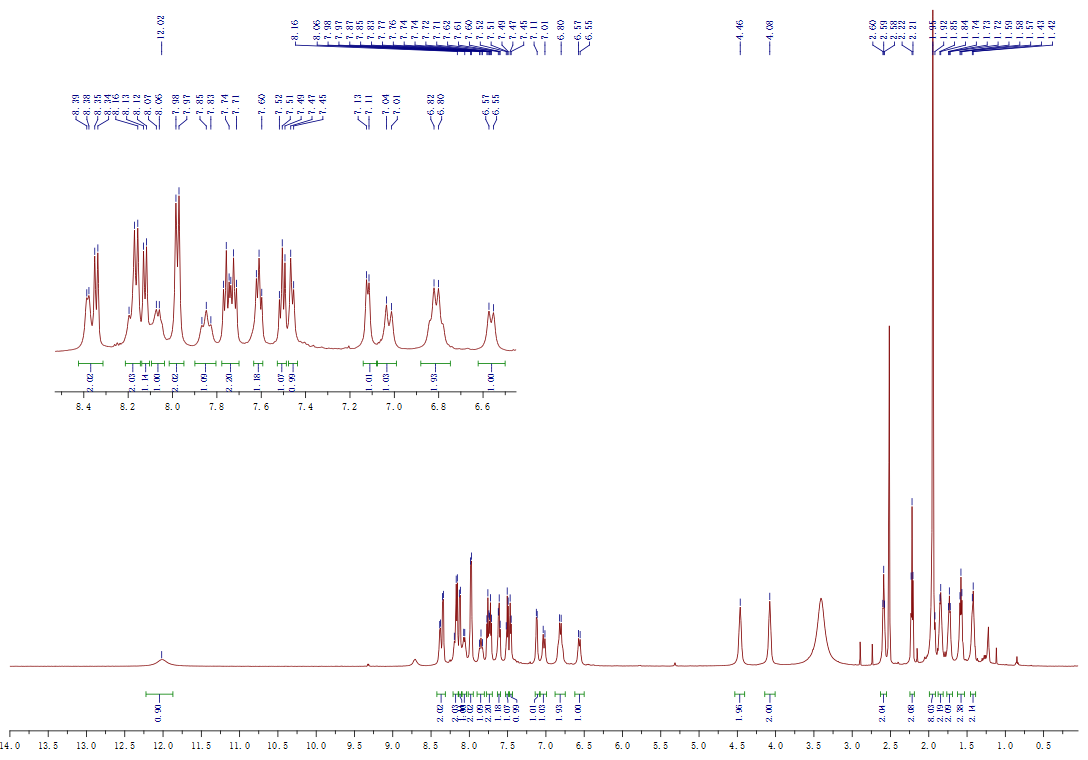


**Figure S11.** ^1^H NMR spectroscopy of compound of **NIC**.


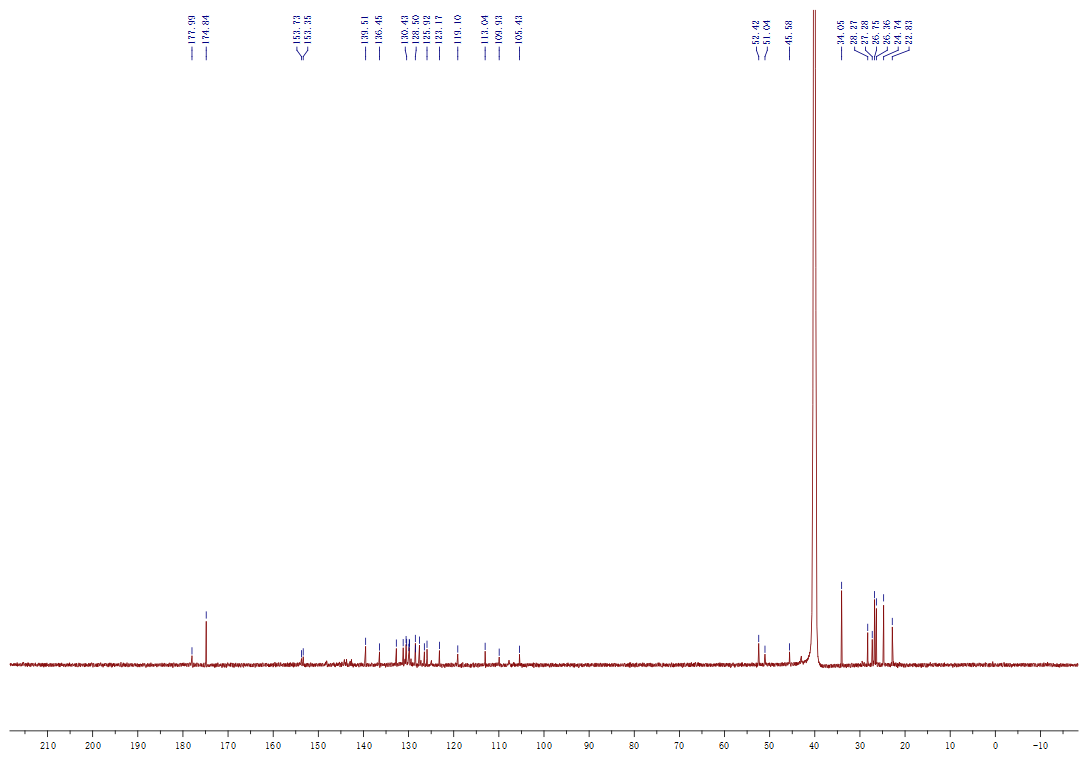


**Figure S12.** ^13^C NMR spectroscopy of compound of NIC.


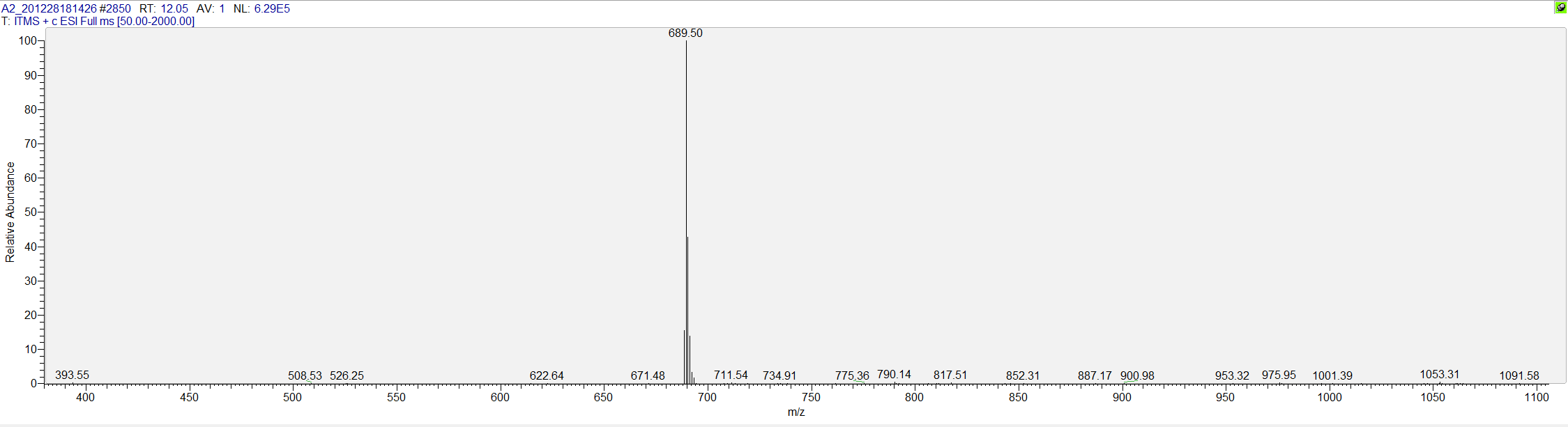


**Figure S13.** MS spectroscopy of **NIC**.


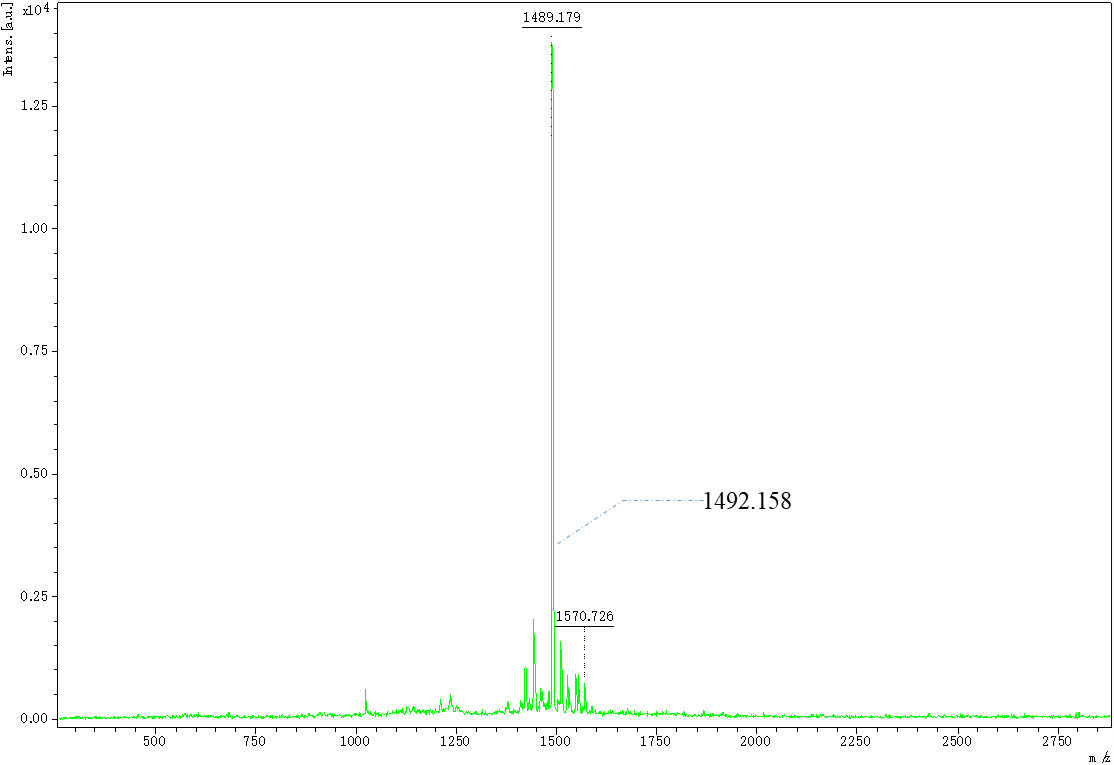


**Figure S14.** MALDI-TOF-MS measurement of **NIC-MLEB**.

**Figure S15.** MALDI-TOF-MS measurement of **NIC-ER**.
